# HIV-Associated Vacuolar Myelopathy and HIV-Associated Dementia as the Initial Manifestation of HIV/AIDS

**DOI:** 10.1155/2019/3842425

**Published:** 2019-09-15

**Authors:** Natalia Wuliji, Matthew J. Mandell, Jason M. Lunt, Adam Merando

**Affiliations:** ^1^Internal Medicine Residency Training Program, Department of Internal Medicine, Saint Louis University School of Medicine, Saint Louis, MO, USA; ^2^Internal Medicine Residency Program at University of Illinois at Chicago/Advocate Christ Medical Center, Oak Lawn, IL, USA

## Abstract

HIV-associated vacuolar myelopathy (HIV-VM) is the most common cause of spinal disease in HIV/AIDS. HIV-VM causes progressive spastic paraparesis, sensory ataxia, and autonomic dysfunction. It is a progressive myelopathy that shares features with subacute combined degeneration seen in vitamin B12 deficiency as well as other neurological diseases and can occur synchronously with HIV-associated dementia (HAD). Here, we describe a rare case in which a patient's initial presentation of HIV/AIDS was both HIV-VM and HAD. A fifty-three-year-old man presented with a six-month history of numerous falls due to progressive gait instability with associated memory loss, tremor, urinary retention, and impotence. His exam was significant for hyperreflexia and weakness in bilateral lower extremities, upgoing plantar reflex, dysmetria, and ataxic gait. MRI-brain was notable for nonspecific volume loss and diffusely increased T2 signal throughout the supratentorial white matter. Lumbar puncture showed isolated lymphocytic pleocytosis with all other CSF testing unremarkable. He ultimately tested positive for HIV-1, with a CD4 count of 157 cells/mm^3^ and a viral load of 874,000 copies/mL. He was diagnosed with HIV-VM and HAD which improved after several months of antiretroviral therapy. This case highlights the importance of considering HIV testing in a patient with a sensory neuropathy and/or progressive cognitive impairment.

## 1. Introduction

HIV-associated vacuolar myelopathy (HIV-VM) is the most common cause of spinal disease in HIV/AIDS [1, 2]. HIV-VM causes progressive spastic paraparesis, sensory ataxia, and autonomic dysfunction [3, 4]. It is characterized pathologically by white matter vacuolization of the posterior and lateral columns of the thoracic spinal cord and less commonly involves the cervical or lumbar spinal cord. It is a progressive myelopathy, sharing features with subacute combined degeneration, seen in vitamin B12 deficiency [1, 2]. It usually presents late in HIV infection—when CD4 counts are very low—and is rarely the presenting symptom of HIV/AIDS [2, 4]. HIV-VM tends to manifest slowly over several months and is often subtle at first [2]. It is a diagnosis of exclusion, as noninfectious and infectious causes of myelopathy must first be ruled out. Typical symptoms include lower extremity weakness; unsteady, stiff, or uncoordinated gait; urinary incontinence or retention; and erectile dysfunction [1]. Historically—in the pre-antiretroviral treatment era—it was identified in AIDS patients as a progressive disease, with death occurring within several months of diagnosis [5]. It can occur synchronously with HIV-associated dementia (HAD) [5]. While treatment is primarily supportive [1], a few case reports have demonstrated improvement of HIV-VM with institution of highly active antiretroviral therapy (HAART) [4, 6] or intravenous immunoglobulin [1].

## 2. Case Presentation

A 53-year-old heterosexual married Caucasian male presented to the neurology clinic with a six-month history of numerous falls, gait instability, impotence, memory loss, tremor, and urinary retention. His sensory ataxia gradually progressed until he required the use of a walker and ultimately became wheelchair-dependent. During this time, he had also suffered from progressive short-term memory loss characterized as difficulty remembering the days of the week, getting lost while driving, and resulted in lost employment as a tree-cutter. Of note, three months prior to establishing care with neurology, the patient established care with a primary care provider with chief complaint of falls. The falls were initially attributed to orthostatic hypotension. He had no documented motor or sensory deficits. HIV and hepatitis screening tests were ordered, but the patient refused. The patient's wife described his mood as “irritable” at times. A standardized assessment of mental status was not obtained. His medical history was remarkable for BPH and osteoarthritis. Physical examination showed pathologically brisk reflexes in the lower extremities with upgoing plantar reflex on the right, dysmetria, a slightly kinetic tremor in bilateral upper extremities, and a wide-based ataxic gait. Vibratory sense was intact. Romberg's sign was positive. His muscle strength was normal in proximal and distal upper and lower extremities initially, but over the course of several months, he developed moderate symmetric weakness in the lower extremities. CT-head was normal. An MRI-brain demonstrated nonspecific volume loss and diffusely increased T2 signal throughout the supratentorial white matter. A cervical spine MRI demonstrated degenerative disease and moderate central canal stenosis; however, patient had no neck, arm, or radicular pain, and Lhermitte's sign was absent. Neurosurgery did not think the MRI findings could explain his profound symptoms and attributed the patient's signs and symptoms to a sensory ataxia.

An extensive infectious workup was obtained. Blood, sputum, and cerebrospinal fluid cultures were negative for aerobic, anaerobic, fungal, and mycobacterial infections. Serologies for endemic mycoses—*Aspergillus*, *Blastomyces*, *Coccidioides*, and *Histoplasma*—were negative. Serologies for *Toxoplasma* and cryptococcal infections were negative. Serologies for syphilis—including the rapid plasma reagin (RPR) and *Treponema pallidum* particle agglutination (TP-PA)—were negative. Hepatitis B and C serologies were negative. Serologies for Lyme disease were not indicative of acute disease (negative Lyme IgM, but positive Lyme IgG antibody). A heavy metal panel was normal. Serum thyroid studies were normal (TSH 1.224 *μ*IU/mL and free T4 0.92 ng/dL). A serum protein electrophoresis was consistent with an inflammatory pattern, but no paraprotein was present. Serum vitamin B12 (511 pg/mL) and folate (14.7 ng/mL) were normal. Cerebral spinal fluid studies for *Toxoplasma*, *Cryptococcus*, *Haemophilus influenzae*, *Listeria monocytogenes*, *Neisseria meningitidis*, *Streptococcus pneumoniae*, JC virus, HTLV-I/II, *Enterovirus*, CMV, HSV-1, HSV-2, HHV-6, and VZV were all negative. CSF was notable for lymphocytic pleocytosis (CSF WBC 34/*μ*L; nl 0–8; 89% lymphocytes), and >5 gamma-restricted oligoclonal bands were present. CSF protein was elevated (179.6 mg/dL; nl 15–40).

The patient denied risk factors for HIV infection, stating he was in a monogamous heterosexual relationship. However, he revealed later that he had high-risk sexual encounters when he was divorced from his wife prior to getting remarried. The patient tested positive for HIV-1 subtype B, with a CD4 count of 157 cells/mm [3]. His viral load was 874,000 copies/mL. Genotypic resistance testing for NRTIs, NNRTIs, PIs, and integrase inhibitors was negative. Patient was started on tenofovir alafenamide, emtricitabine, and dolutegravir.

The patient was seen in the infectious diseases clinic 5 months after starting therapy. His CD4 count was up to 267 cells/mm^3^ and viral load down to 70 copies/mL. He was walking independently without the assistance of a walker or cane although he still required support over longer distances. He continued to have memory problems, but in general was doing much better at remembering daily events and details. His primary complaint was persistent urinary retention, requiring an indwelling catheter.

## 3. Discussion

**Figure 1:**
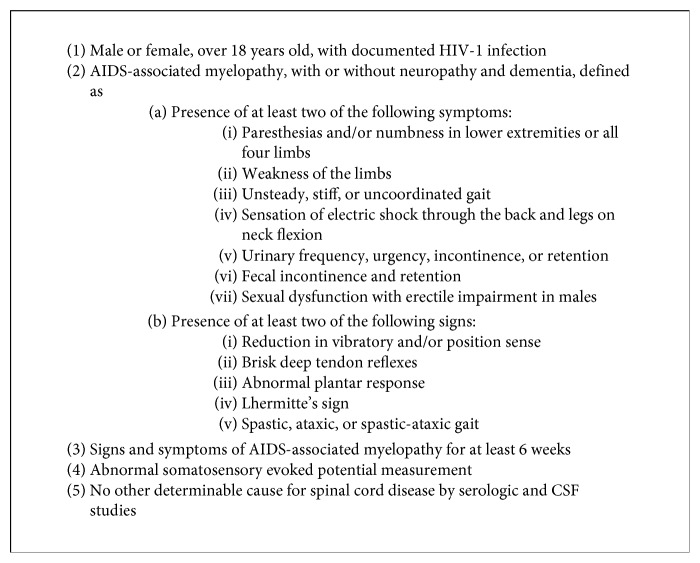
Criteria for clinical diagnosis of AIDS-associated myelopathy [7].

HIV-VM as the presentation of HIV/AIDS is uncommon. While HIV-VM can present at any stage of HIV infection, it is most commonly seen in advanced—and consequently established—HIV disease [1–5, 7]. There are only a handful of case reports which describe HIV-VM as the initial presenting manifestation of HIV/AIDS [3, 4, 6]. While the prevalence of asymptomatic vacuolar myelopathy may be as high as 55% in AIDS patients, the prevalence of symptomatic HIV-VM appears to be much lower [7]. In a study conducted by Petito et al., only 26.8% of AIDS patients with autopsy-proven vacuolar myelopathy had signs and symptoms of the neurologic condition [5]. The diagnosis of HIV-VM is a diagnosis of exclusion, therefore requiring an evaluation for many other etiologies. Criteria for the clinical diagnosis of AIDS-associated myelopathy—adapted from Chong et al.—are shown in Figure 1 [7].

Furthermore, classic findings seen on MRI can help to support the diagnosis of HIV-VM. MRI findings include spinal cord atrophy (nearly universal) and intrinsic cord abnormality, showing increased signal on T2-weighted images in the white matter usually affecting the dorsal columns and lateral corticospinal tracts of the cervical and thoracic cord [7–9]. Of note, three out of the twenty-one patients in Chong et al.'s 1999 study—diagnosed with AIDS-associated myelopathy—had a normal appearing cord on MRI [7].

There are many similarities between the presentation of HIV-VM and subacute combined degeneration of the spinal cord from vitamin B12 deficiency. Both have clinical presentations that include ataxic gait and sensory dysfunction of the extremities and feature vacuolization of the spinal cord white matter due to intramyelin sheath swelling on histology [9]. For this reason, measurement of serum vitamin B12 level (and serum methylmalonic acid and homocysteine in the appropriate clinical setting) is important in differentiating these diagnoses.

Serologic and CSF studies must be performed to rule out alternative causes for CNS disease. The following studies should be obtained: HTLV-I serology, CSF PCR for herpesvirus, and CSF testing for syphilis, CMV, cryptococcal antigens, *Borrelia burgdorferi*, and *Toxoplasma* antibody. A limitation in our case was that CSF analysis for *Borrelia burgdorferi* was not obtained. Our patient otherwise had a negative serologic and CSF workup for alternative causes of CNS disease. Despite our patient's uncharacteristic MRI findings, showing only increased T2 signal throughout the supratentorial white matter with a lack of spinal cord changes, his clinical picture as well as his improvement on HAART favors the diagnosis of HIV-VM.

Our patient presented with both HIV-VM and HAD as the initial manifestation of his HIV/AIDS. Interestingly, HIV-VM and HIV-associated dementia frequently progress in parallel [10]. Similar pathophysiologic mechanisms may underlie HIV-VM and HAD. HIV seems to act differently in the brain—infecting macrophages—rather than T cells as seen in the blood. Despite HAART, HIV seems to have a tropism for perivascular macrophages in the brain where the virus can replicate. One of the reasons why the virus can thrive in the brain may be due to the blood-brain barrier and decreased ability of HAART to be effective in the brain. It has been postulated that HIV enters the CNS early after infection, yet productive infection does not occur until significant immunosuppression develops [10]. HIV likely infects monocytes in the peripheral blood, and as HIV/AIDS progresses, an increased proportion of these circulating activated monocytes are trafficked into the CNS [10].

HAD is thought to be the presenting clinical manifestation of HIV disease in 4–15% of patients with HIV-associated neurocognitive disorders (HAND) [11]. The annual incidence of HAD in HIV patients prior to HAART was 7% [11]. HAD has been characterized as a subcortical dementia, leading to cognitive, behavioral, and motor dysfunction [10, 12]. Initial symptoms may be subtle and are often overlooked or misdiagnosed as depression, [10] as was the case with our patient, whose wife complained of his irritability and whose doctors initially attributed his symptoms to depression. The typical cognitive deficits are characterized primarily by (1) memory loss that is selective for impaired retrieval; (2) impaired ability to manipulate acquired knowledge; (3) personality changes that are characterized by apathy, inertia, and irritability; and (4) general slowing of all thought processes [10]. Motor dysfunction may manifest early on as frequent stumbling and tripping and has been theorized to be due to dopaminergic dysfunction [10]. The diagnosis of HIV-associated dementia is based on the following: (i) progressive cognitive impairment (with or without motor dysfunction) and (ii) exclusion of CNS opportunistic infections and tumors (by CSF and CT/MRI) and is supported by (1) high levels of HIV RNA in the CSF (above 3 log copies/ml) and (2) diffuse, bilateral (often symmetrical) nonenhancing white matter hyperintensities on MRI [12]. Our patient met the above criteria for the diagnosis of HAD. In regard to CSF findings, the CSF is characteristically bland in HIV-VM [8]. However, CSF pleocytosis, while nonspecific, is common in HAD and this was seen in our patient [13]. CSF protein is commonly elevated in patients not on HAART [13].

The prognosis of HIV-associated dementia has historically been poor, with a mean survival of less than 1 year in untreated patients [14]. However, in the era of HAART, we have seen reversal of neurologic deficits and prolonged survival [10]. It is thought that the ability of HAART to reduce HIV CSF viral load correlates with clinical improvement and prolonged survival. Likewise, an undetectable plasma HIV viral load has been shown to attenuate or even halt neurocognitive decline in patients with HAND [14, 15].

## 4. Conclusion

Cognitive impairment and gait ataxia are rare presenting symptoms of HIV/AIDS [16]. This case demonstrates the importance of obtaining a thorough social history with special attention to drug use and sexual practices. In addition to other routine testing for thyroid dysfunction and vitamin deficiencies, HIV should be considered for patients with slowly progressive spastic paraparesis, loss of vibratory and position sense, neurogenic bladder, and in males, erectile dysfunction since initiation of HAART can halt and, in some cases, reverse disease progression and dramatically decrease morbidity and mortality [2].

## Abbreviations

HAART: Highly active antiretroviral therapy
HAD: HIV-associated dementia
HAND: HIV-associated neurocognitive disorders
HIV-VM: HIV-associated vacuolar myelopathy.
